# Hearing rehabilitation in Treacher Collins Syndrome with bone anchored hearing aid

**DOI:** 10.1016/j.rpped.2015.01.010

**Published:** 2015

**Authors:** José Fernando Polanski, Anna Clara Plawiak, Angela Ribas

**Affiliations:** a Faculdade Evangélica do Paraná, Curitiba, PR, Brazil; b Universidade Federal do Paraná (UFPR), Curitiba, PR, Brazil; c Universidade Tuiuti do Paraná (UTP), Curitiba, PR, Brazil

**Keywords:** Mandibulofacial dysostosis, Hearing loss/rehabilitation, Child

## Abstract

**Objective:**

To describe a case of hearing rehabilitation with bone anchored hearing aid in a patient with Treacher Collins syndrome.

**Case description:**

3 years old patient, male, with Treacher Collins syndrome and severe complications due to the syndrome, mostly related to the upper airway and hearing. He had bilateral atresia of external auditory canals, and malformation of the pinna. The initial hearing rehabilitation was with bone vibration arch, but there was poor acceptance due the discomfort caused by skull compression. It was prescribed a model of bone-anchored hearing aid, in soft band format. The results were evaluated through behavioral hearing tests and questionnaires Meaningful Use of Speech Scale (MUSS) and Infant-Toddler Meaningful Auditory Integration Scale (IT-MAIS).

**Comments:**

The patient had a higher acceptance of the bone-anchored hearing aid compared to the traditional bone vibration arch. Audiological tests and the speech and auditory skills assessments also showed better communication and hearing outcomes. The bone-anchored hearing aid is a good option in hearing rehabilitation in this syndrome.

## Introduction

The Treacher Collins syndrome, first described in 1900 by a British optician Edward Treacher Collins, is an autosomal dominant disorder that affects one in 50,000 live births.^1^

In this syndrome, there is a mutation in the *TCOF1* (5q32 locus) gene, responsible for encoding the nucleolar phosphoprotein *Treacle*, which is directly involved with the development of the first two pharyngeal arches.^2^^,^ 3 Approximately 60% of cases do not have a positive family history, but are due to a *de novo* mutation.^4^

The phenotype of these patients is diverse. There are cases in which the patient is mildly affected, and there may be a difficulty in establishing the diagnosis; on the other hand, some patients have early death in the perinatal period, usually caused by the severe airway impairment.^3^ Among the main malformations found are the downward-slanting of palpebral fissure or antimongoloid inclination, mandibular hypoplasia, ear, external auditory canal and middle ear deformities, cleft palate and choanal atresia, among others.^5^

Between 30% and 50% of the affected children have severe bilateral conductive hearing loss, as a result of stenosis or atresia of the external auditory canal or middle ear malformations.^6^ Hearing rehabilitation should be performed as early as possible, in order to ensure the adequate development of language and learning.^1^Because of the severe malformations affecting the patients’ hearing, surgical reconstruction does not usually have good results.^6^

**Figure 1:**
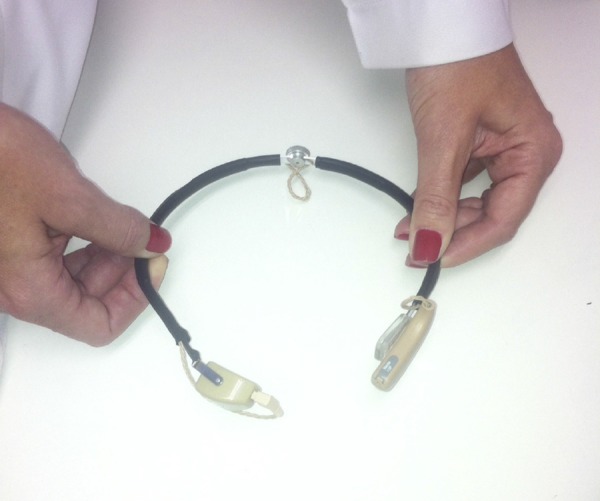
Type of prosthesis.

As a result of the malformation of the external auditory canal, the use of traditional hearing aids, can rarely be indicated. The most common alternative for rehabilitation is the bone vibration arch. This type of prosthesis has some disadvantages, mainly related to excess pressure of the arch, poor esthetical appearance and difficulty in maintaining the arch in place when the patient is a child, as it can be easily removed (Fig. 1).^7^

A recent alternative for the auditory rehabilitation of these patients is the Bone Anchored Hearing Aid (BAHA), which consists of an option in cases of conductive or mixed hearing loss and has a low rate of complications associated with good functional results.^1^

The present study reports the use of BAHA in hearing rehabilitation of a child with Treacher Collins syndrome with bilateral atresia of the external auditory canal. We also describe the methods used in the auditory evaluation and rehabilitation result measurement for patients with this syndrome and at this age group.

## Case report

Case report based on medical file review, approved by the institution's ethics committee under number 24692213.7.0000.0103 and with Informed Consent form signed by the patient's guardian.

The patient was born on September 2010, was male, Caucasian, born and living in Curitiba, Paraná. He was diagnosed with the Treacher Collins syndrome at birth. He had a downward-slanting of palpebral fissure or antimongoloid inclination, malar hypoplasia, micrognathia and macrostomia. He also had microtia and external auditory canal atresia bilaterally.

**Figure 2:**
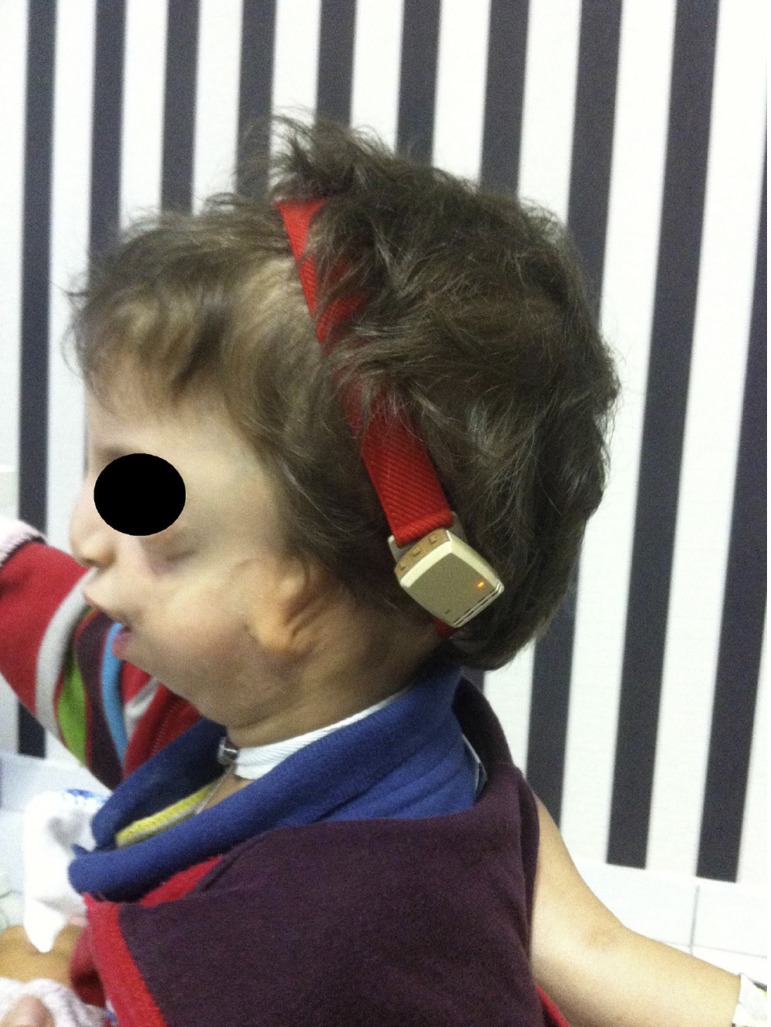
Blepharoplasties.

The most severe complications associated with the syndrome were in the upper airways and the hearing impairment. Tracheostomy was performed soon after birth, and gastrostomy at 3 months of age. Orthognathic surgeries were performed at 1 year and 3 months, 1 year and 8 months and 3 years and 10 months. He is currently stable and well adapted regarding the airways. Additionally, blepharoplasties were performed at 6 months, 8 months, 1 year and 8 months and 1 year and 10 months (Fig. 2).

At the hearing assessment performed on April 2012, at 1 year and 7 months, the objective and subjective tests were used, as described below:

- Brainstem Auditory Evoked Potential: the child had absent airway thresholds in 90dBA and present bone thresholds in 35dBA, on both sides;
- Behavioral note: this test provides clues and information on global development and, especially, the expected auditory behavior for the age of the child, and helps in the diagnosis of hearing loss and other associated disorders.^8^ At the first examination, as shown in Table 1, the child showed lack of response to low and medium intensity sounds (rattle - 50dB; rattle - 70dB; bell - 82dB), presence of auditory attention and search of the sound source for high intensity sounds (castanets - 92dB; agogo bells - 100dB) and absence of cochleopalpebral reflex to high intensity sound (110dB).
  **Table 1:** Behavioral responses without amplification.

Uncalibrated sounds	O	CPR	S	A	SS	LS	LD	LU
50dB rattle	X							
75dB rattle	X							
82dB bell	X							
92dB black–black			X					
100dB large agogo bells			X					
110dB drum			X					
Calibrated sounds	500Hz	1000Hz	2000Hz	4000Hz				
Right ear	80	80	↓	↓				
Left ear	80	80	↓	↓				

O, No response; CPR, cochleopalpebral reflex; S, startled response; A, attention; SS, seeks source; LS, lateralizes to the side; LD, lateralizes downward; LU, lateralizes upward.

After these initial assessments, on August 2012, at 1 year and 10 months, a hearing aid with bone vibrator was adapted. At the test, a functional gain of 40dB was recorded (Table 2); however, device acceptance was poor due to the compression of the skull, and the patient discontinued its use. As a result, the use of a bone-anchored hearing aid was indicated. On August 2013, at 2 years and 10 months, the BAHA system model BP100 with softband was activated and adapted. The behavioral observation recorded functional gain of 60dB (Table 3).

**Table 2:** Behavioral responses with amplification through hearing aid with bone vibration arch.

Uncalibrated sounds	O	CPR	S	A	SS	LS	LD	LU
50dB rattle	X							
75dB rattle			X					
82dB bell			X					
92dB black–black			X					
100dB large agogo bells			X					
110dB drum			X					
Calibrated sounds	500Hz	1000Hz	2000Hz	4000Hz				
Right ear	40	40	60	↓				
Left ear	40	60	60	↓				

O, no response; CPR, cochleopalpebral reflex; S, startled response; A, attention; SS, seeks source; LS, lateralizes to the side; LD, lateralizes downward; LU, lateralizes upward.

**Table 3:** Behavioral responses with amplification through bone-anchored hearing aid.

Uncalibrated sounds	O	CPR	S	A	SS	LS	LD	LU
50dB rattle				X				
75dB rattle				X				
82dB bell				X				
92dB black–black				X				
100dB large agogo bells				X				
110dB drum				X				
Calibrated sounds	500Hz	1000Hz	2000Hz	4000Hz				
Right ear	20	20	20	40				
Left ear	20	20	30	40				

O, no response; CPR, cochleopalpebral reflex; S, startled response; A, attention; SS, seeks source; LS, lateralizes to the side; LD, lateralizes downward; LU, lateralizes upward.

During consultation one month after the BAHA system activation, we applied two protocols to measure the speech and listening skills: Meaningful Use of Speech Scale (MUSS) and Infant-Toddler Meaningful Auditory Integration Scale (IT-MAIS).^9^^,^ 10 Regarding MUSS, which consists of a structured interview with the parents aimed to evaluate the use of speech in everyday situations, the mother was able to identify improvement in the child's communicative intent, including speaking small isolated words. The hearing capacities were analyzed and measured by the IT-MAIS, which consists of a structured interview with the parents, in order to assess the child's spontaneous responses to the sounds in his daily life environment. The child reached, one month after the use of BAHA, a score of 100% in the IT-MAIS, managing to direct attention to the sound source, detect and recognize verbal sounds and react to complex orders.

At an assessment on October 2014, at 4 years of age, during a hearing perception test with no visual cues, he attained the results shown in Table 4. The child remains in speech therapy for auditory and language stimulation, in addition to a multidisciplinary medical follow-up.

**Table 4:** Auditory perception comparing no amplification and bone-anchored hearing aid (BAHA).

Hearing skill	No amplification	With BAHA
*Detection*		
A	12%	100%
I	12%	100%
U	0	100%
Ch	0	100%
Sss	0	100%
Mmm	12%	100%
Discrimination	25%	85%
Closed set	42%	100%
Sentences in a closed set	20%	100%
Sentences in an open set	0	100%

## Discussion

Cases of external auditory canal malformation or atresia often remain without adequate hearing rehabilitation, or end up being rehabilitated in an unsatisfactory manner, with the use of bone vibration arches. These arches, the most often used way to provide rehabilitation in these cases, are frequently poorly accepted by the user, due to esthetic reasons or mainly due to excessive compression on the skin. In addition to the structural issue of this equipment, they have older and limited audiological technology, promoting hearing gains that are often ineffective.

The first models of BAHA became commercially available abroad on 1987.^11^ In Brazil, its use is more recent.^12^ As the system directly stimulates the cochlea without involving the air conduction hearing, i.e. the external auditory canal and middle ear, it is in an excellent option for patients with deformities of the hearing system.

In the studied case, due to the child's young age, we chose to use the equipment in its softband format, which is an option for using the same device with an elastic band. As the child grows and skull thickness consequently increases, the same equipment can be used, but attached to a titanium implant that is surgically inserted into the skull bone. Generally, the adequate bone thickness to receive this implant should be approximately 5 mm, which is the thickness attained at around 5 years of age.^13^ It is known that the implant fixation failure rate is higher in children younger than 5 years.^13^ In addition, patients with Treacher Collins syndrome have delayed growth of the skull bones, which may further accentuate the difficulty of fixing the implants.^14^ The surgical procedure is planned, in this case, to be performed after 5 years of age, when the implant that will be used to attach the same sound processor, previously used in softband format, will be inserted.

The rehabilitated individual shows excellent adaptation to the current method, both regarding the acceptability of the device and the audiological gains provided by it. The measurement of these audiological gains provided by BAHA use through objective auditory tests is not feasible, due to the child's young age - hence the performance of behavioral tests and speech and hearing capacity protocols.^9^^,^ 10

We consider this report to be important, as there are few studies in our country using this technology in hearing rehabilitation. In addition, as far as the literature search demonstrated, no study in our language on the use of such equipment, specifically for auditory rehabilitation in Treacher Collins syndrome, was identified. In the international literature, the studies are not very numerous either.^1^^,^ 13 ^-^ 19 On the other hand, these children, given the degree of multiple disorders, are always followed by multidisciplinary teams, in which the pediatrician is the head of the group and often the main responsible for decision-making and treatment plan. Thus, it is important for everyone involved in the care of these patients to acquire information on these new technologies.

As our final considerations, we reaffirm the importance of disseminating knowledge about the use of this equipment, as it is a new and effective alternative to auditory rehabilitation. Patients with Treacher Collins syndrome comprise a group of individuals that can largely benefit from the use of this technology.

## References

[1] Marsella P, Scorpecci A, Pacifico C, Tieri L (2011). Bone-anchored hearing aid (Baha) in patients with Treacher Collins
syndrome: tips and pitfalls. Int J Pediatr Otorhinolaryngol.

[2] Jensen-Steed G (2011). Treacher Collins syndrome: a case review. Adv Neonatal Care.

[3] Dixon J, Trainor P, Dixon MJ (2007). Treacher Collins syndrome. Orthod Craniofac Res.

[4] Shete P, Tupkari JV, Benjamin T, Singh A (2011). Treacher Collins syndrome. J Oral Maxillofac Pathol.

[5] Thompson JT, Anderson PJ, David DJ (2009). Treacher Collins syndrome: protocol management from birth to
maturity. J Craniofac Surg.

[6] Lesinska E, Stankeviciute V, Petrulionis M (2012). Application of the Vibrant Soundbridge middle-ear implant for aural
atresia in patients with Treacher Collins syndrome. J Laryngol Otol.

[7] Håkansson B, Tjellström A, Rosenhall U (1984). Hearing thresholds with direct bone conduction versus conventional
bone conduction. Scand Audiol.

[8] Azevedo MF (1995). Desenvolvimento auditivo de crianças normais e de alto risco.

[9] Nascimento LT (1997). Uma proposta de avaliação da linguagem oral (Monografia).

[10] Fortunato-Tavares T, Befi-Lopes D, Bento RF, Andrade CR (2012). Children with cochlear implants: communication skills and quality of
life. Braz J Otorhinolaryngol.

[11] Tietze L, Papsin B (2001). Utilization of bone-anchored hearing aids in children. Int J Pediatr Otorhinolaryngol.

[12] Pedriali IV, Buschle M, Mendes RC (2011). Implanted prosthetics osseous conduction (BAHA): reported
cases. Arq Int Otorrinolaringol.

[13] McDermott AL, Williams J, Kuo M, Reid A, Proops D (2009). The birmingham pediatric bone-anchored hearing aid program: a 15 year
experience. Otol Neurotol.

[14] Zeitoun H, De R, Thompson SD, Proops DW (2002). Osseointegrated implants in the management of childhood ear
abnormalities: with particular emphasis on complications. J Laryngol Otol.

[15] Ramakrishnan Y, Marley S, Leese D, Davison T, Johnson IJ (2011). Bone-anchored hearing aids in children and young adults: the Freeman
Hospital experience. J Laryngol Otol.

[16] Habal M, Frans N, Zelski R, Scheuerle J (2003). Percutaneous bone-anchored hearing aid. J Craniofac Surg.

[17] Granström G, Jacobsson C (1999). First and second branchial arch syndrome: aspects on the
embryogenesis, elucidations, and rehabilitation using the osseointegration
concept. Clin Implant Dent Relat Res.

[18] Van der Pouw KT, Snik AF, Cremers CW (1998). Audiometric results of bilateral bone-anchored hearing aid application
in patients with bilateral congenital aural atresia. Laryngoscope.

[19] Thomas J (1996). Speech and voice rehabilitation in selected patients fitted with a
bone anchored hearing aid (BAHA). J Laryngol Otol Suppl.

