# Supplementary material for: Hearing rehabilitation in Treacher Collins Syndrome with bone anchored hearing aid
Source: Rev Paul Pediatr. 2015 Oct-Dec;33(4):483–7. doi: 10.1016/j.rpped.2015.01.010 (PMC4685570; doi:10.1016/j.rpped.2015.01.010)
Supplement: Translation

# Translation: Reabilitação auditiva na Síndrome de Treacher Collins por meio de prótese auditiva ancorada no osso

**Authors:** José Fernando Polanski, Anna Clara Plawiak, Angela Ribas

**Affiliations:** a Faculdade Evangélica do Paraná, Curitiba, PR, Brasil; b Universidade Federal do Paraná (UFPR), Curitiba, PR, Brasil; c Universidade Tuiuti do Paraná, Curitiba, PR, Brasil

**Keywords:** Disostose mandibulofacial, Deficiência auditiva/reabilitação, Criança

## Abstract

**Objetivo:**

Descrever um caso de reabilitação auditiva por meio do uso de prótese auditiva ancorada no osso em um paciente portador da síndrome de Treacher Collins.

**Descrição do caso:**

Paciente de três anos, masculino, portador da síndrome de Treacher Collins, com complicações graves relacionadas à síndrome, principalmente de vias aéreas altas e audição. Apresentava atresia de condutos auditivos externos bilateralmente, além de malformação dos pavilhões auditivos. Para reabilitação auditiva inicial foi indicado aparelho auditivo em arco de vibração óssea, porém houve péssima aceitação pelo desconforto causado pela compressão no crânio. Foi indicado como método opcional um modelo de prótese auditiva ancorada no osso, no formato *softband*. Os resultados foram avaliados por meio de testes auditivos comportamentais e dos questionários Meaningful Use of Speech Scale (Muss) e Infant-Toddler Meaningful Auditory Integration Scale (IT-Mais).

**Comentários:**

O paciente teve uma excelente aceitação da prótese auditiva ancorada no osso quando comparada com o tradicional arco de vibração óssea. Os testes audiológicos, bem como as avaliações de habilidades de fala e de audição, também demonstraram melhores capacidades de comunicação e audição. Esse equipamento mostra-se uma boa opção na reabilitação auditiva de portadores dessa síndrome.

## Introdução

A síndrome de Treacher Collins, descrita pela primeira vez em 1900 pelo oftalmologista britânico Edward Treacher Collins, é um distúrbio autossômico dominante que afeta um a cada 50.000 nascidos vivos.^1^

Nessa síndrome há uma mutação no gene *TCOF1* (locus 5q32), responsável por codificar a fosfoproteína nucleolar *Treacle*, que está diretamente envolvida com o desenvolvimento dos dois primeiros arcos faríngeos.^2^^,^ 3 Aproximadamente 60% não têm história familiar positiva, mas sim devem-se a uma mutação de novo.^4^

O fenótipo dos pacientes acometidos é variado. Há casos em que o paciente é levemente afetado e pode haver uma dificuldade de estabelecimento do diagnóstico; por outro lado, alguns pacientes têm morte precoce, no período perinatal, causada geralmente pelo grande comprometimento das vias aéreas.^3^ Dentre as principais malformações encontradas estão a inclinação da fissura palpebral inferior ou inclinação antiongoloide, hipoplasia mandibular, deformidades auriculares, no conduto auditivo externo e na orelha média, fenda palatina, atresia de coana, entre outras.^5^

Entre 30% e 50% das crianças acometidas apresentam perda auditiva condutiva bilateral de grau severo devido a estenose ou atresia do conduto auditivo externo ou por malformações da orelha média.^6^ A reabilitação auditiva deve ser feita o mais precocemente possível, com o objetivo de permitir um bom desenvolvimento da linguagem e do aprendizado.^1^ Devido às malformações graves que acometem o aparelho auditivo dos portadores, a reconstrução cirúrgica não costuma apresentar bons resultados.^6^

Já as malformações do conduto auditivo externo fazem com que o uso de aparelhos auditivos tradicionais, os aparelhos de amplificação sonora individuais (AASI), raramente possam ser indicados. A opção de reabilitação mais comum é prótese auditiva de vibração óssea, acoplada a um arco. Essa prótese apresenta uma série de inconvenientes, principalmente relacionados à pressão excessiva do arco, à estética ruim e à dificuldade de se manter o arco em crianças, por ser facilmente removível (Fig. 1).^7^

**Figura 1:** Modelo de prótese.

Uma opção recente para reabilitação auditiva desses pacientes é a prótese auditiva ancorada no osso (PAAO), que consiste em uma opção em casos de perdas condutivas ou mistas que apresenta um baixo índice de complicações associado a bons resultados funcionais.^1^

O presente estudo relata o uso de PAAO na reabilitação auditiva de uma criança portadora da síndrome de Treacher Collins, com atresia bilateral de condutos auditivos externos. Descrevemos também os métodos de avaliação auditiva e de mensuração de resultados da reabilitação, para pacientes com essa síndrome e nessa faixa etária.

## Descrição do caso

Relato de caso baseado em revisão de prontuário aprovado pelo comitê de ética da instituição sob o número 24692213.7.0000.0103 e com Termo de Consentimento Livre e Esclarecido assinado pela responsável pelo paciente.

Paciente nascido em 23/09/10, masculino, branco, natural e residente em Curitiba, PR. Diagnosticado como portador da síndrome de Treacher Collins ao nascimento. Apresentava inclinação antimongoloide das fissuras palpebrais, hipoplasia malar, micrognatia e macrostomia. Contava ainda com microtia e atresia de conduto auditivo externo bilateralmente.

As complicações mais graves relacionadas à síndrome eram as das vias aéreas altas e audição. Foi feita traqueostomia logo após o nascimento e gastrostomia aos três meses. Cirurgias ortognáticas foram feitas com um ano e três meses, um ano e oito meses e aos três anos e 10 meses. Atualmente, encontra-se estável e com boa adaptação no que diz respeito às vias respiratórias. Além disso, blefaroplastias foram feitas aos seis meses, oito meses, um ano e oito meses e um ano e 10 meses (Fig. 2).

**Figura 2:** Blefaroplastia.

Na avaliação auditiva feita em 25/04/12, com um ano e sete meses, foram aplicados testes objetivos e subjetivos, conforme descritos a seguir:

- Potencial evocado auditivo de tronco encefálico: a criança apresentou limiares de via aérea ausentes em 90dBA e limiares de via óssea presentes em 35dBA em ambos os lados;
- Observação comportamental: esse teste fornece pistas e informações sobre o desenvolvimento global e principalmente o comportamento auditivo esperado para faixa etária da criança, além de auxiliar no diagnóstico da deficiência auditiva e de outros distúrbios associados.^8^ No primeiro exame feito na criança, como mostrado na Tabela 1, registrou-se a ausência de resposta para sons de fraca e média intensidade (guizo-50dB; chocalho-70dB; sino-82dB), presença de atenção auditiva e procura da fonte sonora para sons de forte intensidade (castanhola-92dB; agogô-100dB) e ausência de reflexo cócleo-palpebral para som de forte intensidade (110dB).
  **Tabela 1:** Respostas comportamentais sem amplificação

Sons não calibrados	O	RCP	S	A	PF	LL	LB	LC
50dB guizo	X							
75dB guizo	X							
82dB sino	X							
92dB black‐black			X					
100dB agogô grande			X					
110dB tambor			X					
Sons calibrados	500Hz	1000Hz	2000Hz	4000Hz				
Orelha direita	80	80	↓	↓				
Orelha esquerda	80	80	↓	↓				

O, Sem resposta; RCP, Reflexo cócleo‐palpebral; S, sobressalto; A, atenção; PF, procura a fonte; LL, lateraliza para o lado; LB, lateraliza para baixo; LC, lateraliza para cima.

Após essas avaliações iniciais, em 14/08/12, com um ano e 10 meses, foi adaptada uma prótese auditiva com vibrador ósseo. No teste foi registrado ganho funcional de 40dB (Tabela 2), porém a aceitação do aparelho foi péssima, pela compressão no crânio, e o paciente descontinuou o uso. Em função disto, foi indicado o uso de uma prótese auditiva ancorada no osso. Em 20/08/13, aos dois anos e 10 meses, o sistema Baha modelo BP100, com *softband*, foi ativado e adaptado. A observação comportamental registrou ganho funcional de 60dB (Tabela 3).

**Tabela 2:** Respostas comportamentais com amplificação por meio de prótese auditiva com vibrador ósseo

Sons não calibrados	O	RCP	S	A	PF	LL	LB	LC
50dB guizo	X							
75dB guizo			X					
82dB sino			X					
92dB black‐black			X					
100dB agogô grande			X					
110dB tambor			X					
Sons calibrados	500Hz	1000Hz	2000Hz	4000Hz				
Orelha direita	40	40	60	↓				
Orelha esquerda	40	60	60	↓				

O, Sem resposta; RCP, Reflexo cócleo‐palpebral; S, sobressalto; A, atenção; PF, procura a fonte; LL, lateraliza para o lado; LB, lateraliza para baixo; LC, lateraliza para cima.

**Tabela 3:** Respostas comportamentais com amplificação por meio de prótese auditiva ancorada no osso

Sons não calibrados	O	RCP	S	A	PF	LL	LB	LC
50dB guizo				X				
75dB guizo				X				
82dB sino				X				
92dB black‐black				X				
100dB agogô grande				X				
110dB tambor				X				
Sons calibrados	500Hz	1000Hz	2000Hz	4000Hz				
Orelha direita	20	20	20	40				
Orelha esquerda	20	20	30	40				

O, Sem resposta; RCP, Reflexo cócleo‐palpebral; S, sobressalto; A, atenção; PF, procura a fonte; LL, lateraliza para o lado; LB, lateraliza para baixo; LC, lateraliza para cima.

Em consulta feita um mês após a ativação do sistema Baha, aplicaram-se dois protocolos para mensurar as habilidades de fala e de audição: Meaningful Use of Speech Scale (Muss) e Infant-Toddler Meaningful Auditory Integration Scale (IT-Mais).^9^^,^ 10 Ao Muss, que consiste em uma entrevista estruturada com os pais com o objetivo de avaliar o uso de fala em situações cotidianas, a mãe conseguiu identificar melhoria na intenção comunicativa da criança, inclusive com emissão de pequenas palavras isoladas. As habilidades auditivas foram analisadas e mensuradas pelo IT-Mais, que consiste em uma entrevista aos pais estruturada com o objetivo de avaliar as respostas espontâneas da criança para os sons em seu ambiente de vida diário. A criança atingiu, um mês após o uso da PAAO, 100% de escore no IT-Mais e conseguiu direcionar a atenção à fonte sonora, detectar e reconhecer sons verbais e reagir a ordens complexas.

Numa avaliação em 01/10/14, aos quatro anos, num teste de percepção auditiva sem pista visual, obteve-se o resultado demonstrado na Tabela 4. A criança continua em atendimento fonoaudiológico para estimulação auditiva e de linguagem, além do acompanhamento médico multidisciplinar.

**Tabela 4:** Percepção auditiva comparada sem amplificação e com PAAO

Habilidade auditiva	Sem amplificação	Com PAAO
*Detecção*		
A	12%	100%
I	12%	100%
U	0	100%
Ch	0	100%
Sss	0	100%
Mmm	12%	100%
Discriminação	25%	85%
Conjunto fechado	42%	100%
Frases em conjunto fechado	20%	100%
Frases em conjunto aberto	0	100%

## Discussão

Casos de malformação ou atresia do conduto auditivo externo muitas vezes permanecem sem reabilitação auditiva adequada ou acabam reabilitados de maneira pouco satisfatória com o uso de arcos de vibração óssea. Esses arcos, a maneira de reabilitação mais usada nesses casos, muitas vezes são pouco aceitos pelo usuário por questões estéticas ou, principalmente, por compressão excessiva sobre a pele. Além da questão estrutural desses equipamentos, eles apresentam tecnologia audiológica mais antiga e limitada e promovem ganhos audiológicos muitas vezes pouco efetivos.

Os primeiros modelos de PAAO passaram a ser comercialmente disponíveis no exterior em 1987.^11^ No Brasil o seu uso é mais recente.^12^ Como o sistema estimula diretamente a cóclea, sem envolver a condução auditiva aérea, ou seja, o meato acústico externo e a orelha média, consiste em uma excelente opção para pacientes com deformidades do aparelho auditivo.

No caso em questão, pela baixa idade da criança optou-se pelo uso do equipamento no seu formato *softband*, que é uma opção de uso do mesmo aparelho por meio de uma faixa elástica. Com o crescimento da criança e consequentemente aumento da espessura da calota craniana, o mesmo equipamento pode ser usado, mas acoplado a um implante de titânio cirurgicamente inserido no osso do crânio. Geralmente, a espessura adequada do osso para receber esse implante deve ser de aproximadamente 5 mm, espessura essa que é adquirida por volta dos cinco anos.^13^ Sabe-se que o índice de falha da fixação do implante é maior em crianças com menos de cinco anos.^13^ Além disso, portadores da síndrome de Treacher Collins apresentam crescimento atrasado dos ossos cranianos, o que pode acentuar ainda mais a dificuldade de fixação dos implantes.^14^ Planeja-se, no caso em questão, procedimento cirúrgico a partir dos cinco anos, quanto se irá inserir o implante que servirá para acoplar o mesmo processador de som previamente usado no formato *softband*.

O indivíduo reabilitado apresenta excelente adaptação ao método atual, tanto na aceitabilidade do dispositivo quanto dos ganhos audiológicos por ele propiciados. A mensuração desses ganhos audiológicos por meio de testes auditivos objetivos em uso da PAAO não são viáveis pela baixa idade da criança, daí a aplicação de testes comportamentais e protocolos de habilidade de fala e audição.^9^^,^ 10

Vemos como importante este relato pelo fato de ainda haver poucos estudos, em língua portuguesa, com o uso dessa tecnologia na reabilitação auditiva. Além disso, até onde se estendeu o levantamento bibliográfico, nenhum estudo na nossa língua foi encontrado com o uso desse equipamento especificamente na reabilitação auditiva na síndrome de Treacher Collins. Na literatura internacional eles também não são muito numerosos.^1^^,^ 13 ^-^ 19 Por outro lado, essas crianças, dado o grau de múltiplos acometimentos, são sempre acompanhadas por equipes multidisciplinares nas quais o pediatra é o centro do grupo e muitas vezes o principal responsável por orientar as decisões e condutas. Por isso é importante que as informações sobre essas novas tecnologias sejam de amplo conhecimento de todos os envolvidos no atendimento a esses pacientes.

Como considerações finais, reafirma-se a importância da difusão do conhecimento sobre o uso desse equipamento, por ser ele uma nova e eficaz opção de reabilitação auditiva. Pacientes portadores da síndrome de Treacher Collins se constituem num grupo que pode ser muito beneficiado com o uso dessa tecnologia.
